# Real-world effectiveness and safety of tofacitinib and abatacept in patients with rheumatoid arthritis

**DOI:** 10.1093/rap/rkac090

**Published:** 2022-10-29

**Authors:** Wataru Hirose, Masayoshi Harigai, Koichi Amano, Toshihiko Hidaka, Kenji Itoh, Kazutoshi Aoki, Masahiro Nakashima, Hayato Nagasawa, Yukiko Komano, Toshihiro Nanki

**Affiliations:** Hirose Clinic of Rheumatology, Saitama, Japan; Division of Rheumatology, Department of Internal Medicine, Tokyo Women’s Medical University School of Medicine, Tokyo, Japan; Department of Rheumatology and Clinical Immunology, Saitama Medical Center, Saitama Medical University, Saitama, Japan; Institute of Rheumatology, Zenjinkai Miyazaki-Zenjinkai Hospital, Miyazaki, Japan; Division of Rheumatology, Department of Internal Medicine, National Defense Medical College, Saitama, Japan; Aoki Clinic of Rheumatology, Saitama, Japan; Department of Immunology and Microbiology, National Defense Medical College, Saitama, Japan; Nagasawa Clinic of Rheumatology,, Saitama, Japan; Division of Rheumatology, Department of Internal Medicine, Jujo Takeda Rehabilitation Hospital, Kyoto, Japan; Division of Rheumatology, Department of Internal Medicine, Toho University School of Medicine, Tokyo, Japan

**Keywords:** RA, shared epitope, tofacitinib, abatacept, inverse probability of treatment weighting

## Abstract

**Objective:**

We compared the 52-week effectiveness and safety of tofacitinib (TOF) and abatacept (ABT) in patients with RA in a real-world setting and investigated a role of human leucocyte antigens (HLA)-DRB1 shared epitope (SE) in the effectiveness.

**Methods:**

RA patients starting TOF (*n* = 187) and ABT (*n* = 183) were enrolled. Effectiveness was compared after reducing the selection bias to a minimum using the inverse probability of treatment weighting (IPTW) based on propensity scores. The influence of SE alleles on effectiveness was compared within each treatment group. A treatment group comparison was also performed within SE-positive and SE-negative groups.

**Results:**

Herpes zoster and some laboratory abnormalities were more frequent in the TOF group than in the ABT group. Patient characteristics did not differ significantly between treatment groups after adjustments with IPTW. The TOF group had a significantly higher proportion of DAS in 28 joints using ESR (DAS28-ESR) remission at week 52 than the ABT group. The DAS28-ESR at week 12 and thereafter was not affected by the copy number of SE alleles in the TOF group, but decreased significantly as the copy number increased in the ABT group. In SE-positive patients, remission and drug retention rates did not differ significantly between the two treatment groups. In SE-negative patients, the TOF group showed significantly higher remission and drug retention rates than the ABT group.

**Conclusion:**

The present results suggest that TOF is more effective with regard to remission at week 52 based on treatment responses in SE-negative RA patients.

Key messages

- Tofacitinib was superior to abatacept regarding remission at week 52 after adjustments with inverse probability of treatment weighting.
- Abatacept was as effective as tofacitinib for shared epitope-positive RA and less effective for shared epitope-negative RA.
- Herpes zoster and some laboratory abnormalities were more frequent with tofacitinib than with abatacept.

## Introduction

The introduction of biologic DMARDs (bDMARDs) and targeted synthetic DMARDs has significantly improved the outcome of RA. The 2020 update guideline of the Japan College of Rheumatology recommended the use of bDMARDs or Janus kinase (JAK) inhibitors with or without MTX in phase II (i.e. after initial treatment with MTX or conventional synthetic DMARDs (csDMARDs) [1]. Tofacitinib (TOF) is an oral JAK inhibitor that preferentially reduces signalling from type I and II cytokine receptors by inhibiting JAK 3 and/or JAK 1 [2, 3]. Abatacept (ABT) is a genetically engineered fusion protein that selectively inhibits T-cell activation by binding to CD80/86 and modulating their interactions with CD28 [4]. The efficacies of TOF and ABT have been confirmed in patients with RA receiving monotherapy, MTX-inadequate responders (IRs) and TNF inhibitor-IRs in randomized controlled trials [3–8]. However, head-to-head trials to compare TOF with ABT have not yet been conducted. Therefore, in our previous report, we compared the 24-week effectiveness of TOF and ABT in RA patients using propensity score matching [9].

Among susceptibility genes to RA, the strongest relationship was reported with the HLA region, particularly *HLA-DRB1* alleles that share a similar amino acid sequence, called the shared epitope (SE) [10]. Although the SE hypothesis was initially proposed to explain genetic susceptibility to RA, subsequent investigations suggested that the primary role of SE is in the development of more severe disease manifestations [11, 12]. Autoantibodies, such as ACPA, are more likely to occur in SE-positive RA patients [13–15]. SE has also been linked to progressive joint damage [16]. Furthermore, SE might affect responses to treatment [17–19], and we previously confirmed the impact of SE on responses to TOF and ABT within each treatment group [9].

In the present study, we compared the 52-week clinical outcomes of TOF and ABT using the inverse probability of treatment weighting (IPTW) based on a propensity score that reduces the selection bias to a minimum and adjusts for confounding factors between binary treatment groups. We also investigated the effects of SE positivity on clinical outcomes in each treatment group and between treatment groups.

## Methods

### Patients and study design

This was a multicentre, longitudinal observational study conducted at 12 hospitals and clinics for rheumatology in Japan. We enrolled patients aged ≥20 years who fulfilled the 2010 ACR/EULAR classification criteria for RA [20] and started treatment with TOF or ABT between January 2015 and January 2021. The prior use of bDMARDs or JAK inhibitors did not limit patient enrolment in the present study. Data from the patients who started treatments with TOF or ABT between January 2015 and December 2017 were obtained retrospectively from the patients’ medical records, whereas data from the patients who started the treatment between January 2018 and January 2021 were obtained prospectively. The data collection schedule is presented in Supplementary Table S1, available at *Rheumatology Advances in Practice* online. An *HLA-DRB1* allele analysis was performed at enrolment. Written informed consent was obtained according to the Declaration of Helsinki. This study design was initially approved by the Ethics Committee of Toho University School of Medicine (approved number, A19062_A18107_A17085), then by each participating centre or institution. This study was registered with the University Hospital Medical Information Network Clinical Trial Registry (UMIN000037418).

### Treatment with TOF and ABT

TOF or ABT was administered to RA patients in whom disease activity was not controlled by MTX or csDMARDs or to RA patients unable to be treated with csDMARDs, including MTX. The dosage of TOF was adjusted by renal function. Patients with an estimated glomerular filtration rate >60 ml/min/1.73 m^2^ received 5 mg of TOF orally twice daily, whereas those with estimated glomerular filtration rate <60 ml/min/1.73 m^2^ received 5 mg of TOF orally once daily. ABT was administered as an i.v. infusion (500 mg for patients weighing <60 kg, 750 mg for 60–100 kg, and 1000 mg for >100 kg) at weeks 0, 2 and 4, then every 4 weeks thereafter. Alternatively, patients received 125 mg by s.c. injection once weekly [21].

### Clinical effectiveness and outcome

Disease activity was assessed by the 28-joint count DAS using the ESR (DAS28-ESR) [22], the simplified disease activity index (SDAI) [23] and the clinical disease activity index (CDAI) [24] at baseline and after 4, 12, 26 and 52 weeks. The primary outcome was the remission rate at week 52 in each group, measured by DAS28-ESR. DAS28-ESR remission was defined as a score of <2.6 and low disease activity (LDA) as a score of <3.2. Additional secondary outcomes included disease activity, the retention rate and safety at week 52.

### *HLA-DRB1* genotyping and autoantibody detection

The *HLA-DRB1* allele was genotyped by the SeCore DRB1 Locus Exon 2 & 3 Sequencing kit (One Lambda, CA, USA) with the PCR sequencing-based typing method. HLA-DRB1*01:01, *04:01, *04:04, *04:05, *04:10, *10:01, *14:02 and *14:06 were defined as SE [9]. ACPA was detected using a second-generation anti-CCP kit (Abbott Japan Laboratories, Tokyo, Japan). A cut-off value of 4.5 U/ml was used for anti-CCP antibody positivity.

### Safety

The incidence and severity of all adverse events were recorded until week 52. The common terminology criteria for adverse events of the National Cancer Institute (v.5.0) were used to describe and grade adverse events and laboratory abnormalities.

### Adjustment with IPTW for a comparison of clinical outcomes between TOF and ABT groups

IPTW based on propensity scores was applied to adjust for the baseline characteristics of patients receiving TOF and ABT. Weights were calculated for each individual as 1/propensity score for the ABT group and 1/(1 − propensity score) for the TOF group. The propensity score was calculated using the logistic regression as the probability of being treated with ABT against TOF. After adjustments by IPTW, the effectiveness of TOF and ABT was compared, and predictors leading to DAS28-ESR remission were analysed in each treatment group. When the effectiveness of TOF and ABT was compared with SE-positive and SE-negative patients separately, adjustments by IPTW based on propensity scores that differed from those used in the analysis for overall patients were applied. Details of the procedure for calculating propensity scores are shown in Supplementary Data S1, and Supplementary Table S2 shows the logistic regression model used for the estimation of propensity scores (Supplementary material is available at *Rheumatology Advances in Practice* online).

### Other statistical analyses

Differences between groups for normally distributed continuous data were examined using Student’s unpaired *t*-test. Pearson’s χ^2^ test was used for categorized variables. The weighted Kaplan–Meier method was used to assess retention rates, and treatment group differences were analysed by the IPTW log-rank test [25]. A multivariable logistic regression analysis was performed to identify factors contributing to DAS28-ESR remission at week 52 in each treatment group. Explanatory variables were age, male sex, the duration of RA, HAQ-DI, bDMARD naïve, DAS28-ESR at baseline, SE positive and anti-CCP antibody positivity. Values of *P* < 0.05 were considered significant. The last observation carried forward method was used for patients who discontinued treatment before week 52 to include all patients in the analysis. All statistical analyses were performed with R v.3.6.1 (R Core Team, 2019, Vienna, Austria).

## Results

### Comparison of safety in TOF and ABT groups

Overall, 187 RA patients starting TOF and 183 starting ABT between January 2015 and January 2021 were enrolled and followed up for 52 weeks. Table 1 shows the adverse events observed during the 52 weeks before adjustments with IPTW. No significant differences were observed in the incidence of any adverse events, as specified by the common terminology criteria for adverse events, or serious adverse events and infections that might lead to the discontinuation of the two drugs between the two treatment groups. The incidence of herpes zoster was significantly higher in the TOF group than in the ABT group (9.1 *vs* 2.7%, *P* = 0.014). Three patients, including one case of grade 3, discontinued TOF prematurely owing to herpes zoster. No significant differences were noted in the incidence of cancer or major adverse cardiovascular and venous thromboembolic events between the two treatment groups. Laboratory data showed a significant decrease in the neutrophil count in both groups and a significant increase in the lymphocyte count in the ABT group. Low-density lipoprotein (LDL) cholesterol, high-density lipoprotein (HDL) cholesterol and creatinine phosphokinase (CPK) levels were significantly elevated in the TOF group. No cases showed grade 3 or 4 increases in CPK levels in either group.

**Table 1.** Safety and laboratory data, weeks 0–52

Parameter	Tofacitinib	Abatacept	*P*-value
	(*n* = 187)	(*n* = 183)	
Event	Adverse events to week 52		

Any adverse event, *n* (%)	130 (69.5)	124 (67.8)	0.74
Serious adverse event, *n* (%)	14 (7.5)	9 (4.9)	0.31
Death	0 (0)	0 (0)	1.00
Serious infection, *n* (%)	4 (2.1)	4 (2.2)	1.00
Herpes zoster, *n* (%)	17 (9.1)	5 (2.7)	0.014
Cancer, *n* (%)	0 (0)	1 (0.5)	1.00
MACE, *n* (%)	2 (1.1)	1 (0.5)	0.49
VTE, *n* (%)	0 (0)	1 (0.5)	0.49

Variables	Change in laboratory values from baseline to week 52		

Haemoglobin, g/dl	0.15 (−0.04, 0.34)	0.58 (0.39, 0.78)	0.0019
Neutrophils, /µL	−841 (−1152, −530)	−929 (−1249, −608)	0.70
Lymphocytes, /µL	−55 (−149, 50)	265 (157, 373)	3.7×10^−5^
LDL cholesterol, mg/dl	22.1 (17.2, 27.1)	6.1 (0.51, 11.6)	3.0×10^−5^
HDL cholesterol, mg/dl	7.5 (4.9, 10.1)	3.1 (0.2, 6.1)	0.030
ALT, U/l	3.9 (1.0, 6.8)	1.4 (−1.6, 4.3)	0.23
AST, U/l	6.3 (3.5, 9.0)	2.3 (−0.5, 5.0)	0.049
Creatinine, mg/dl	0.05 (0.02, 0.08)	0.07 (0.03, 0.10)	0.60
Creatinine phosphokinase, U/l	70.5 (54.2, 86.8)	1.8 (−17.6, 21.3)	5.1×10^−7^

Adverse events, infection or laboratory abnormalities leading to the permanent discontinuation of tofacitinib or abatacept are designated as serious adverse events. The data shown are the numbers and percentages of patients with adverse events. Laboratory values are reported as the least-squares mean change from baseline at week 52.

ALT: alanine aminotransferase; AST: aspartate aminotransferase; HDL: high-density lipoprotein; LDL: low-density lipoprotein; MACE: major adverse cardiovascular event; VTE: venous thromboembolism.

### Patient characteristics in TOF and ABT groups after adjustments with IPTW

Table 2 (left-hand side) shows patient characteristics before adjustments. Patients in the ABT group were significantly older than those in the TOF group. In addition, the ABT group had significantly higher swollen joint counts, ESR, CRP, HAQ-DI and anti-CCP antibody titres than the TOF group. The ABT group also included significantly more bDMARD-naïve patients than the TOF group. We then calculated IPTW using propensity scores to reduce the selection bias to a minimum and adjusted patient characteristics. Adjusted characteristics are shown in Table 2 (right-hand side). No significant differences were observed in any patient characteristics. The distribution of variables was well balanced.

**Table 2.** Patient characteristics in tofacitinib and abatacept groups before and after inverse probability of treatment weighting

	Before IPTW			After IPTW		
Variable	TOF	ABT	*P*-value	TOF	ABT	*P*-value
	(*n* = 187)	(*n* = 183)		(*n* = 184)	(*n* = 178)	
Age, years	66.2 (11.5)	70.7 (11.3)	1.3×10^−4^	68.5 (10.8)	66.1 (16.1)	0.10
Female, *n* (%)	158 (84.5)	154 (84.2)	1.0	152 (82.3)	152 (85.4)	0.22
Disease duration, years	14.2 (11.5)	14.3 (13.0)	0.55	14.4 (12.6)	14.3 (11.8)	0.92
BMI, kg/m²	22.1 (3.8)	21.9 (3.5)	0.67	21.8 (3.8)	21.8 (3.5)	0.94
SE copy number 0/1/2, %	27.8/61.0/11.2	33.3/52.5/14.2	0.60	24.7/64.1/11.2	30.6/38.6/10.8	0.33
Current smoker, *n* (%)	11 (5.9)	10 (5.5)	1.0	12 (6.5)	10 (5.6)	0.60
Ever smoker, *n* (%)	50 (26.7)	48 (26.2)	1.0	47 (25.5)	47 (26.4)	0.82
MTX use, *n* (%)	109 (58.3)	94 (51.4)	0.22	105 (57.1)	93 (52.2)	0.20
MTX dose, mg/week	8.6 (2.5)	8.2 (2.4)	0.21	8.5 (2.3)	8.9 (3.0)	0.30
Oral CS use, *n* (%)	70 (37.4)	66 (36.1)	0.87	70 (38.0)	63 (35.4)	0.46
Oral CS dose, mg/day^a^	4.4 (2.5)	4.7 (2.8)	0.42	5.0 (2.8)	4.5 (2.9)	0.32
bDMARD naïve, *n* (%)	40 (21.4)	122 (66.7)	4.3×10^−18^	83 (45.1)	80 (44.9)	0.82
Number of biologics previously used, *n* 0/1/2/≥3	40/60/39/48	122/31/12/17	5.9×10^−17^	83/43/25/33	80/38/33/27	0.87
SJC, 0–28	3.8 (3.9)	4.4 (3.7)	0.016	3.9 (3.8)	3.7 (3.5)	0.68
TJC, 0–28	5.4 (4.9)	5.4 (5.0)	0.99	5.4 (4.7)	4.8 (5.1)	0.27
GH, VAS, 0–100 mm	55.0 (27.4)	53.5 (24.7)	0.47	53.0 (27.6)	52.0 (25.0)	0.72
EGA, VAS, 0–100 mm	49.1 (20.7)	43.6 (18.1)	0.013	46.7 (19.3)	44.0 (18.8)	0.18
DAS28-ESR	4.6 (1.5)	4.8 (1.2)	0.081	4.7 (1.4)	4.5 (1.4)	0.26
SDAI	21.4 (11.8)	21.4 (11.0)	0.90	21.1 (11.5)	20.0 (10.9)	0.27
CDAI	19.7 (10.8)	19.4 (9.5)	0.93	19.3 (10.6)	18.3 (9.7)	0.29
ESR, mm/h	36.9 (31.2)	43.0 (29.4)	0.014	39.0 (29.7)	37.8 (29.9)	0.71
CRP, mg/dl	1.7 (2.2)	1.9 (2.4)	0.023	1.8 (2.1)	1.7 (2.3)	0.52
MMP-3, ng/ml	211.0 (219.5)	216.4 (245.6)	0.42	238.7 (247.6)	190.9 (220.4)	0.060
RF positive, *n* (%)	149 (79.7)	152 (83.1)	0.48	144 (78.3)	142 (79.8)	0.58
RF, U/ml	181.2 (372.4)	199.3 (508.6)	0.67	194.5 (455.7)	201.4 (591.8)	0.92
Anti-CCP antibody positive, *n* (%)	163 (87.2)	158 (86.3)	0.94	162 (88.0)	151 (84.8)	0.18
Anti-CCP antibody titre, U/ml	224.0 (310.2)	303.0 (375.5)	0.024	227.6 (324.2)	276.8 (372.0)	0.18
HAQ-DI	0.94 (0.73)	1.1 (0.80)	0.029	1.1 (0.8)	1.0 (0.8)	0.61

Results are expressed as means (s.d.) unless otherwise stated. Comparisons of matched groups were performed using Student’s *t*-test for continuous variables and Pearson’s χ^2^ test for categorized variables.

a Prednisolone equivalents.

ABT: abatacept; CDAI: Clinical Disease Activity Index; DAS28-ESR: DAS in 28 joints using ESR; EGA: evaluator’s global assessment of disease activity; GH: patient’s global assessment of general health; HAQ-DI: HAQ disability index; IPTW: inverse probability of treatment weighting; SDAI: simplified disease activity index; SE: shared epitope; SJC: swollen joint count; TJC: tender joint count; TOF: tofacitinib.

### Adjustment with IPTW for a comparison of clinical outcomes between TOF and ABT groups

**Figure 1.**
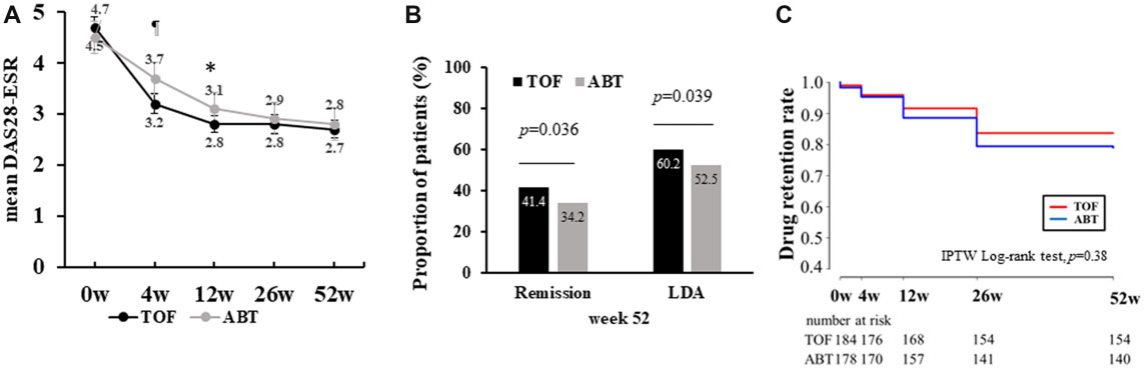
Effectiveness and drug retention rates of tofacitinib and abatacept. (**A**) After adjustments with IPTW, DAS28-ESR between the two groups was compared at each time point. Error bars indicate 95% CIs. *P <0.05, ^¶^*p* <0.01 by Student’s *t*-test. (**B**) The rates of remission and achievement of LDA in DAS28-ESR at week 52 were compared between the two groups by Pearson’s χ^2^ test. (**C**) Drug retention rates in both groups are shown over 52 weeks (Kaplan–Meier curves). ABT: abatacept; DAS28-ESR: DAS in 28 joints using the ESR; IPTW: inverse probability of treatment weighting; LDA: low disease activity; TOF: tofacitinib

Fig. 1 shows effectiveness and retention rates over 52 weeks of treatment with TOF and ABT after adjustments with IPTW. DAS28-ESR, CDAI and SDAI over 52 weeks are shown in Fig. 1A and Supplementary Fig. S1 (available at *Rheumatology Advances in Practice* online). The TOF group had significantly lower CDAI [TOF *vs* ABT; 4.8±4.0 *vs* 5.98±5.4, *P* = 0.049] and SDAI [TOF *vs* ABT; 5.08±4.1 *vs* 6.28±5.6, *P* = 0.035] at week 52, while the difference in DAS28-ESR at week 52 between the two groups [TOF *vs* ABT; 2.78±1.0 *vs* 2.88±1.1, *P *=* *0.35] was not significant. However, the TOF group showed significantly higher rates of DAS28-ESR remission [odds ratio (OR) = 1.38, 95% CI = 1.02, 1.87, *P* = 0.036] and DAS28-ESR-LDA achievement (OR = 1.36, 95% CI = 1.02, 1.83, *P *=* *0.039; Fig. 1B) and had significantly lower DAS28-CRP compared with the ABT group (Supplementary Fig. S2A and B, available at *Rheumatology Advances in Practice* online) at week 52. In addition, the TOF group showed a significantly higher proportion of DAS28-CRP remission (OR = 1.74, 95% CI = 1.30, 2.33, *P* = 2.18 × 10^−14^) and DAS28-CRP LDA achievement (OR = 1.71, 95% CI = 1.26, 2.32, *P* = 5.11 × 10^−14^). The drug retention rate over 52 weeks did not differ significantly between the TOF and ABT groups (TOF *vs* ABT; 83.2 *vs* 80.0%, *P *=* *0.38; Fig. 1C). Supplementary Fig. S3A and B, available at *Rheumatology Advances in Practice* online, summarizes patient disposition flow charts showing the number of patients on drug and who discontinued taking the drug at each observation point, with the reasons for the discontinuation.

### Impact of SE on responses to treatment with TOF and ABT within each treatment group

**Figure 2.**
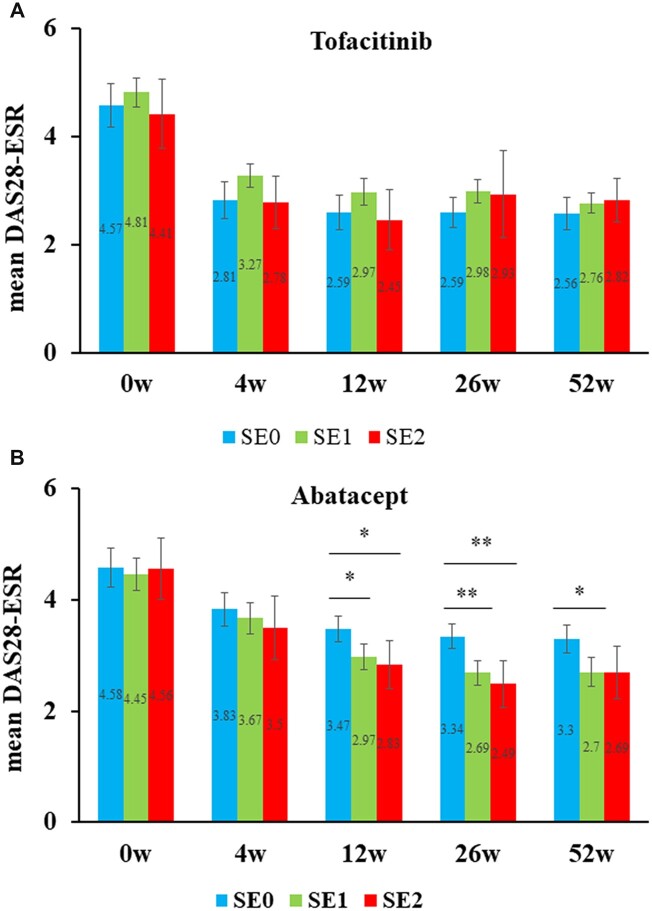
DAS in 28 joints using the ESR based on copy numbers for shared epitope alleles after adjustments with inverse probability of treatment weighting. DAS28-ESR stratified by the copy number of SE alleles (i.e. 0, 1 and 2) is shown over 52 weeks after the introduction of tofacitinib (**A**) or abatacept (**B**). DAS28-ESR was compared between the three groups at each time point within each treatment group by Student’s *t*-test with Bonferroni corrections. Error bars indicate 95% CIs. Values of *P* < 0.0167 are considered to be significant. *P <0.0167; **P <0.0033. DAS-ESR: DAS in 28 joints using the erythrocyte sedimentation rate; IPTW, inverse probability of treatment weighting; SE: shared epitope

The time courses of DAS28-ESR stratified according to the copy numbers of SE alleles after adjustments with IPTW are shown in Fig. 2. The number of cases with each SE allele number was as follows: in the order of zero, one and two SE alleles, 51, 112 and 21 cases in the TOF group and 58, 94 and 26 cases in the ABT group. DAS28-ESR in each treatment group at each time point was compared between zero and one SE allele, zero and two SE alleles, and one and two SE alleles using Student’s *t*-test with Bonferroni corrections, which adjust *P*-values of <0.0167 to be significant. In the TOF group, no significant differences were observed in DAS28-ESR regardless of the copy numbers of SE alleles. In contrast, in the ABT group, significant differences were observed in DAS28-ESR that depended on the copy numbers of SE alleles. The following analyses showed significant differences in DAS28-ESR: zero *vs* one SE allele [3.478±0.85 *vs* 2.978±1.10 *P *=* *0.0048] and zero *vs* two SE alleles [3.478±0.85 *vs* 2.838±1.12, *P* = 0.0057] at week 12; zero *vs* one SE allele [3.348±0.79 *vs* 2.698±1.05, *P* = 2.05 × 10^−4^] and zero *vs* two SE alleles [3.348±0.79 *vs* 2.498±1.08, *P* = 2.12 × 10^−4^] at week 26; and zero *vs* one SE allele [3.258±0.86 *vs* 2.708±1.17, *P* = 0.0075] at week 52. In addition, the proportion of patients achieving DSA28-ESR remission during the 52 weeks did not differ significantly between SE-positive and SE-negative patients in the TOF group, whereas the ABT group showed a significantly higher remission rate in SE-positive patients than in SE-negative patients (Supplementary Fig. S4, available at *Rheumatology Advances in Practice* online).

### Prediction for DAS28-ESR remission

We performed analyses to identify factors associated with DAS28-ESR remission at week 52. Explanatory variables were age, sex, RA disease duration, HAQ-DI, DAS28-ESR, bDMARD naïve, SE-positive and anti-CCP antibody positivity at baseline. In the TOF group, an examination of prognostic factors at baseline contributing to DAS28-ESR remission at week 52 showed that HAQ-DI and bDMARD naïve were independent prognostic factors. In contrast, HAQ-DI, disease duration and the presence of SE alleles and anti-CCP antibody positivity were associated with DAS28-ESR remission after 52 weeks of ABT treatment. The presence of SE alleles and the anti-CCP antibody at baseline was not associated with DAS28-ESR remission in the TOF group (Table 3).

**Table 3.** Independent predictors for remission in DAS in 28 joints using ESR at week 52 by a multivariable logistic regression analysis

Variable	Tofacitinib		Abatacept	
	OR (95% CI)	*P*-value	OR (95% CI)	*P*-value
Age ≥65 years old	1.40 (0.81, 2.44)	0.23	0.64 (0.38, 1.10)	0.11
Sex (male)	2.19 (1.12, 4.25)	0.021	0.49 (0.23, 1.03)	0.062
RA duration	0.99 (0.97, 1.01)	0.4	0.97 (0.94, 0.99)	0.011
HAQ-DI	0.28 (0.19, 0.43)	1.52×10^−9^	0.35 (0.22, 0.55)	6.32×10^−6^
DAS28-ESR	0.83 (0.68, 1.00)	0.055	0.91 (0.72, 1.14)	0.39
bDMARD naïve	2.35 (1.41, 3.91)	9.82×10^−4^	1.14 (0.65, 2.02)	0.64
SE positive	0.84 (0.46, 1.50)	0.55	4.13 (2.08, 8.18)	4.89×10^−5^
Anti-CCP antibody positive	1.69 (0.76, 3.75)	0.20	5.52 (1.87, 16.32)	2.00×10^−3^

Predictive factors for remission in DAS28-ESR at week 52 in patients receiving tofacitinib or abatacept were examined using a multivariable logistic regression model adjusted for age ≥65 years old (yes/no), sex, RA disease duration, DAS28-ESR, HAQ-DI, bDMARD-naïve (yes/no), shared epitope positive (yes/no), and anti-cyclic citrullinated peptide antibody positive (yes/no).

DAS28-ESR: DASe in 28 joints using ESR; HAQ-DI: HAQ Disability Index; SE: shared epitope.

### Adjustment with IPTW for a comparison of clinical outcomes within each SE category

**Figure 3.**
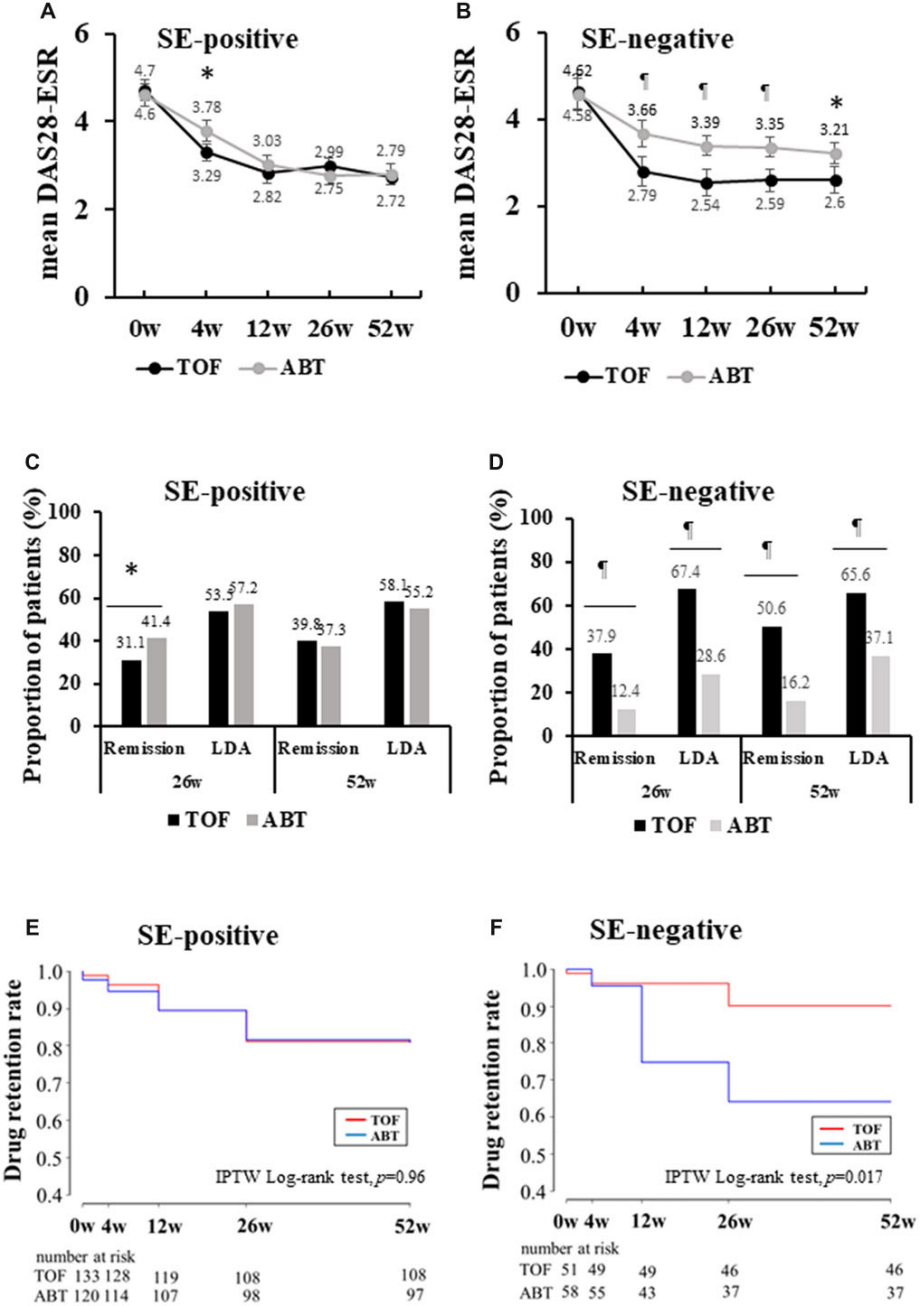
Comparison of tofacitinib and abatacept between shared epitope-positive and -negative patients. DAS28-ESR for SE-positive (**A**) and SE-negative (**B**) patients after adjustments with IPTW are shown after the introduction of TOF and ABT. The rates of remission and achievement of LDA in DAS28-ESR in SE-positive (**C**) and SE-negative (**D**) patients were compared between the two groups by Pearson’s χ^2^ test. Drug retention rates over 52 weeks of TOF and ABT in SE-positive (**E**) and SE-negative (**F**) patients were compared by the weighted Kaplan–Meier method. Error bars indicate 95% CIs. *P <0.05, ^¶^*P* <0.001 by Student’s *t*-test. ABT: abatacept; DAS-ESR: DAS in 28 joints using the erythrocyte sedimentation rate; IPTW, inverse probability of treatment weighting; LDA: low disease activity; SE: shared epitope; TOF: tofacitinib

We adjusted baseline patient characteristics between the two groups within each SE category by IPTW based on propensity scores that differed from those used in the analysis for overall patients. Patient characteristics before and after adjustments are shown in Supplementary Tables S3 and S4, available at *Rheumatology Advances in Practice* online. No significant differences were observed in any patient characteristics in both treatment groups after adjustments with IPTW. The distribution of variables was well balanced. Comparisons of the effectiveness of TOF and ABT were performed at each time point over 52 weeks within SE-positive and SE-negative patients. In SE-positive patients, DAS28-ESR did not differ significantly between the two groups (Fig. 3A). In contrast to SE-positive patients, SE-negative patients had significantly lower DSA28-ESR in the TOF group than in the ABT group throughout the 52-week period (Fig. 3B). Furthermore, the rates of remission and LDA achievement in DAS28-ESR at weeks 26 and 52 did not differ significantly between the two groups in SE-positive patients, except for remission at week 26 (Fig. 3C). In contrast, SE-negative patients receiving TOF had significantly higher rates of remission and LDA achievement in DAS28-ESR at weeks 26 and 52 than those receiving ABT (Fig. 3D). Similar results were observed in the analysis according to CDAI (Supplementary Figure S5, available at *Rheumatology Advances in Practice* online). Treatment group differences for drug retention rates within each SE category were assessed by the weighted Kaplan–Meier method and IPTW log-rank test based on propensity scores. In SE-positive patients, the drug retention rate over 52 weeks did not differ significantly between the two groups (log-rank test, *P *=* *0.96; Fig. 3E), whereas in SE-negative patients, patients receiving TOF had a significantly higher retention rate at week 52 than those receiving ABT (log-rank test, *P *=* *0.017; Fig. 3F).

## Discussion

In the present study, the safety of TOF and ABT for RA patients was recorded for 52 weeks. Although no significant differences were observed in the incidence of adverse events or serious adverse events and infection that might lead to drug discontinuation between the two groups, the incidence of herpes zoster was significantly higher in the TOF group than in the ABT group (9.1 *vs* 2.7%, *P *=* *0.014). In an integrated analysis of randomized controlled trials and long-term extension studies, the incidence per 100 patient-years of herpes zoster was 4.0 in the global RA programme and 8.0 in Japan [26]. It remains unclear whether the increased risk was attributable to genetic, cultural or environmental differences between Japanese and Western populations. The mechanism for the increased risk of herpes zoster might be related to the inhibition of IFN signalling. Antiviral defences rely on type I and II IFN signalling via the JAK/STAT pathway [27], which is inhibited by TOF [28]. Mean changes in laboratory tests from baseline to week 52 showed significantly higher LDL and HDL cholesterol levels in the TOF group than in the ABT group. In phase II and III trials, both doses of TOF (5 or 10 mg twice daily), either as monotherapy or in combination with csDMARDs, were associated with 15–20% increases in LDL and HDL cholesterol levels in comparisons of data obtained 4 weeks after treatment initiation and baseline values [29]. Although recent findings from the ORAL Surveillance post-marketing safety trial have indicated a potentially increased risk of major adverse cardiovascular events with TOF [30], there is no known direct mechanism that explains the detrimental effects of TOF on the risk of cardiovascular outcomes.

Patient characteristics at baseline between the treatment groups were imbalanced in observational studies, hence direct comparisons are inappropriate [31]. Therefore, in the present study, we adjusted baseline patient characteristics using IPTW based on propensity scores. Within the ABT group, the proportion of DAS28-ESR remission at week 52 was significantly higher in SE-positive patients than in SE-negative patients (SE-positive *vs* SE-negative: 41.6 *vs* 15.4%, *P* = 0.00047). In contrast, within the TOF group, the two groups did not show a significant difference (SE-positive *vs* SE-negative: 38.1 *vs* 51.4%, *P* = 0.10; Supplementary Fig. S2, available at *Rheumatology Advances in Practice* online). These results provide important information for the management of RA using these drugs. Namely, TOF is effective for the treatment of RA regardless of the presence or absence of SE, whereas the effectiveness of ABT might be limited in SE-negative patients. The SE alleles cannot be measured in clinical settings and might be replaced with anti-CCP antibody, which is multicollinear with SE in ACPA-positive RA [32]. Anti-CCP antibody titres might be one of the predictive biomarkers for responses to treatment with ABT in patients with seropositive and early RA [33].

Predictors of the response to treatment revealed a marked difference between the two treatment groups. SE and anti-CCP antibody positivity were both identified as significant predictors of responses to treatment with ABT. This might be a characteristic of ABT. Another study reported that higher anti-CCP antibody titres were correlated with better DAS28-CRP responses with ABT [34]. We also found a correlation between the presence of SE and DAS28-ESR remission in patients receiving ABT [9]. In contrast, the present results revealed that neither SE nor anti-CCP antibody positivity affected responses to treatment with TOF. Low HAQ-DI at baseline was associated with DAS28-ESR remission at week 52 in both the TOF and ABT groups. Another study extracted higher HAQ-DI at baseline as a factor for belonging to the treatment-resistant group in patients receiving TOF [35], which is consistent with the present results. In addition, being bDMARD naïve was identified as a predictor of remission in DAS28-ESR in the TOF group. These results are very important for managing RA using TOF, namely, the initiation of TOF at an earlier phase of RA might be more effective. In contrast, in the treatment with ABT, the rate of patients with good/moderate responses was reportedly higher among bDMARD-naïve patients than among those with previous biologic failure [36]. However, in the present study, following the inclusion of SE and anti-CCP antibody positivity in a multivariable logistic analysis, being bDMARD naïve was not a significant predictor of DAS28-ESR remission at week 52 in patients receiving ABT. Our results suggest that ABT is effective in RA patients positive for SE or anti-CCP antibodies and with a short disease duration and low HAQ-DI regardless of the previous use of bDMARDs.

The effectiveness and retention rates of TOF and ABT were compared within each SE category after adjustments for patient characteristics with IPTW. Within SE-positive patients, DAS28-ESR remission and drug retention rates at week 52 did not differ significantly between the two groups. In contrast, within SE-negative patients, remission and drug retention rates at week 52 were significantly higher in patients receiving TOF than in those receiving ABT. These results provide important evidence for the selection of drugs to treat RA. Namely, these two medications exert similar effects for SE-positive patients. However, TOF might be a better choice than ABT for SE-negative patients.

The present study has several limitations. This was an analysis of a small number of Japanese patients, hence our results might not be applicable to all patients with RA. Furthermore, although IPTW based on propensity scores was used to reduce the selection bias to a minimum and adjust for patient characteristics, not all confounding factors were adjusted. There might be unknown confounding factors. Moreover, owing to the small number of patients examined, it was not possible to compare effectiveness and drug retention rates between TOF and ABT within each SE category, which consists of zero, one and two copies of SE alleles. We failed to adjust for the baseline characteristics of patients with two copies of SE alleles using IPTW. Therefore, patients had to be divided into two groups according to the presence or absence of SE alleles. Finally, there is no definitive basic study that supports the difference in efficacy of TOF and ABT between SE-positive and SE-negative patients. Thus, further investigation regarding a comparison of the effects of these drugs via the JAK-STAT signal transduction pathway and T-cell co-stimulatory molecules on the regulation of disease activity of RA will be required.

In conclusion, in RA patients, TOF was superior to ABT with regard to remission at week 52, but was associated with higher frequencies of herpes zoster and laboratory abnormalities. ABT was as effective as TOF in SE-positive patients and less effective in SE-negative patients. Additional data from longer and larger trials are needed to obtain a more detailed understanding of the long-term outcomes and safety of TOF and ABT relative to those of other drugs for the treatment of RA.

## Supplementary data

Supplementary data are available at *Rheumatology Advances in Practice* online.

## Supplementary Material

rkac090_Supplementary_Data

## Data Availability

The data underlying this article are available in the article and its online supplementary material.
