# Supplementary material for: Real-world effectiveness and safety of tofacitinib and abatacept in patients with rheumatoid arthritis
Source: Rheumatol Adv Pract. 2022 Oct 29;6(3):rkac090. doi: 10.1093/rap/rkac090 (PMC9667967; doi:10.1093/rap/rkac090)
Supplement: rkac090_Supplementary_Data [file rkac090_supplementary_data.docx]

***Wataru Hirose et al. Real-world effectiveness and safety of tofacitinib and abatacept in patients with rheumatoid arthritis***

**Supplementary Data S1. Details of the procedure for calculating propensity score.** To calculate the propensity scores, multivariable logistic regression analysis was performed with the use of ABT as the dependent and the following as independent variables; age, sex, disease duration, height, weight, shared epitope (yes/no), rheumatoid factor, C-reactive protein (CRP), erythrocyte sedimentation rate (ESR), number of neutrophils, number of lymphocytes, hemoglobin, albumin, swollen joint count, visual analog scale by evaluator global assessment, Disease Activity Score in 28 joints using ESR (DAS28-ESR), Health Assessment Questionnaire Disability Index (HAQ-DI), biologic disease-modifying antirheumatic drug (bDMARD)-naïve (yes/no), anti-cyclic citrullinated peptide (CCP) antibody positive (yes/no).

**Supplementary Data S2. TOF-ABT study investigators who provided blood samples for the analysis of HLA-DRB1 alleles**

| Yuji Akiyama | Division of Rheumatology, Department of Internal Medicine, Ogawa Red Cross Hospital, Saitama, Japan |
| --- | --- |
| Souichirou Ando | Ando Clinic of Rheumatology, Saitama, Japan |
| Kentarou Chino | Department of Rheumatology and Clinical Immunology, Saitama Medical Center, Saitama Medical University, Saitama, Japan |
| Yayoi Hashiba | Institute of Rheumatology, Zenjinkai Miyazaki-Zenjinkai Hospital,  Miyazaki, Japan |
| Motohide Kaneko | Kaneko Clinic of Rheumatology, Saitama, Japan |
| Mitsuhiro Kawagoe | Aoki Clinic of Rheumatology, Saitama, Japan |
| Tsuneo Kondo | Department of Rheumatology and Clinical Immunology, Saitama Medical  Center, Saitama Medical University, Saitama, Japan |
| Kazuyoshi Kubo | Institute of Rheumatology, Zenjinkai Miyazaki-Zenjinkai Hospital,  Miyazaki, Japan |
| Ikuko Masuda | Jujo Takeda Rehabilitation Hospital, Kyoto, Japan |
| Mitsuyo Matsumoto | Hirose Clinic of Rheumatology, Saitama, Japan |
| Yusuke Okada | Department of Rheumatology and Clinical Immunology, Saitama Medical  Center, Saitama Medical University, Saitama, Japan |
| Akiko Shibata | Department of Rheumatology and Clinical Immunology, Saitama Medical  Center, Saitama Medical University, Saitama, Japan |
| Kimihiro Suzuki | Suzuhiro Clinic of Rheumatology, Saitama, Japan |
| Ko Takamatsu | Division of Rheumatology, Department of Internal Medicine, National Defense, Medical College, Saitama, Japan |
| Hirofumi Takei | Division of Rheumatology, Department of Internal Medicine, Kanoiwa Hospital, Yamanashi, Japan |

**
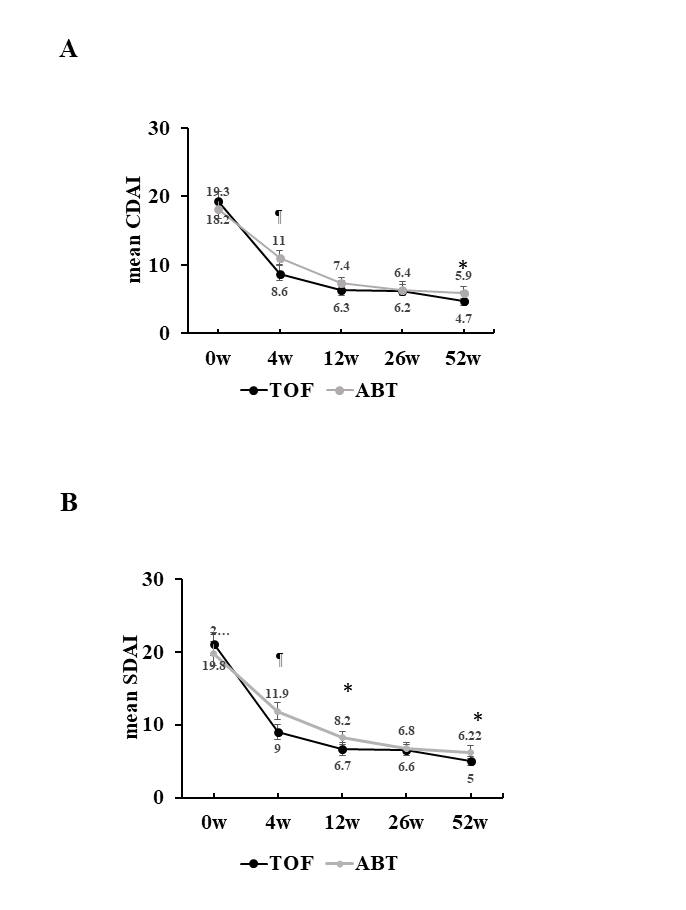
**

**Supplementary Figure S1. Disease activity over 52 weeks in tofacitinib (TOF) and abatacept (ABT) groups.** After adjustments with IPTW based on propensity scores, disease activity scores in CDAI (A) and SDAI (B) between the two treatment groups were compared at each time point. Error bars indicate 95% confidence intervals. * *p*<0.05, ¶ *p*<0.01 by the Student’s *t*-test.

**
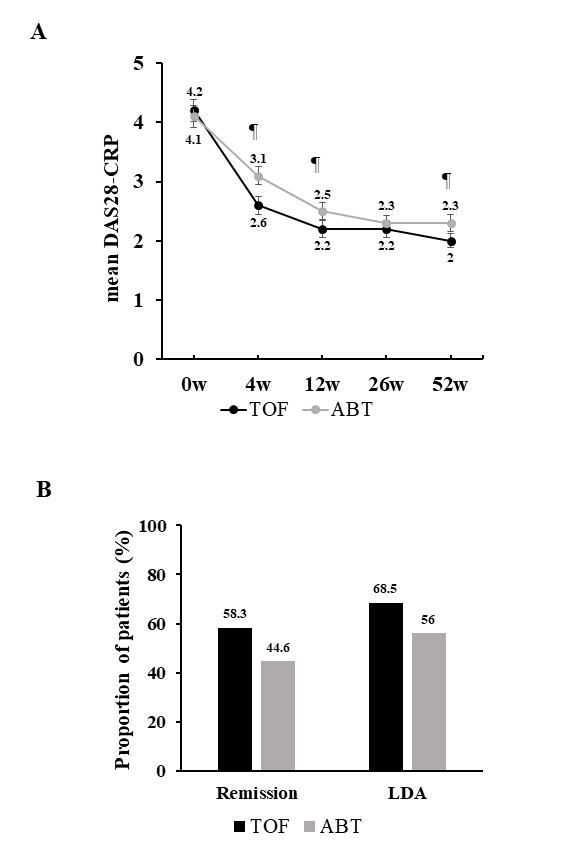
**

**Supplementary Figure S2. Comparison of the effectiveness of TOF and ABT by DAS28-CRP.** (A) After adjustment with IPTW, DAS28-CRP between the two treatment groups was compared at each time point. Error bars indicate 95% confidence intervals. ¶ p< 0.01 by the Student’s *t*-test. (B) The rates of remission and low disease activity (LDA) achievement in DAS2-CRP at week 52 compared between the two treatment groups by Pearson’s χ² test. DAS28-CRP, Disease Activity Score in 28 joints using the C-reactive protein; IPTW, inverse probability of treatment weighting.


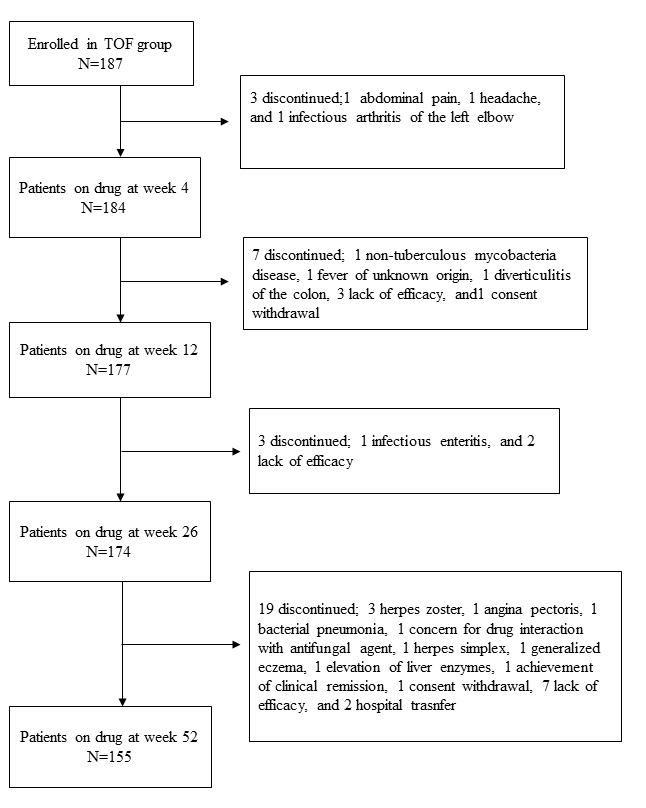


**Supplementary Figure S3A**

**
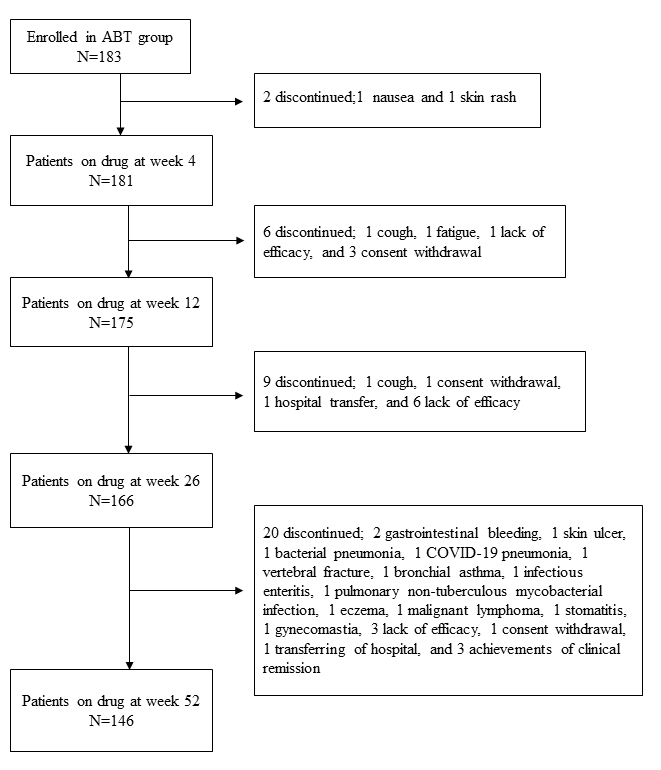
Supplementary Figure S3B**

**Supplementary Figure S3. Patient disposition flow charts for the tofacitinib group (A) and abatacept group (B) from enrollment until 52 weeks.** The reasons for discontinuation described in each flow chart partially overlapped.

**
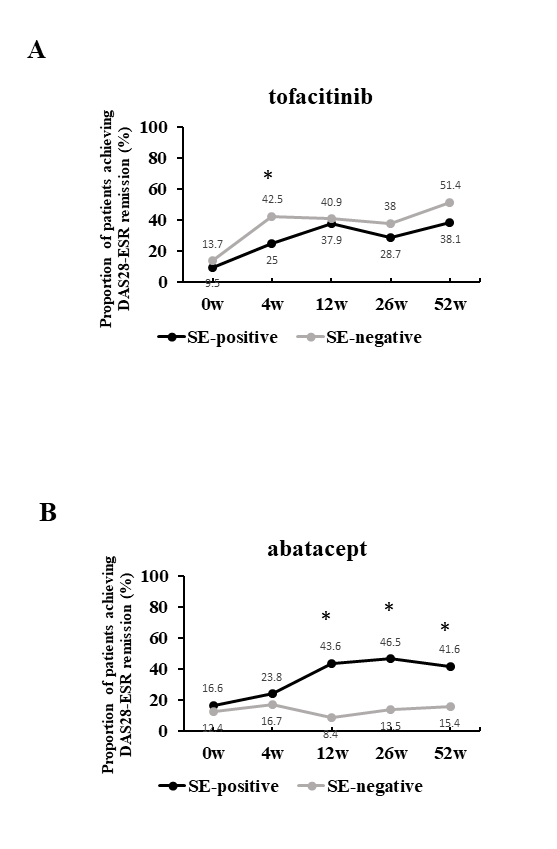
**

**Supplementary Figure S4. Comparison of the DAS28-ESR remission rate within each treatment group.** The proportions of patients achieving DAS28-ESR remission in the tofacitinib (A) and abatacept (B) groups were compared at each time point between SE-positive and SE-negative patients over 52 weeks after adjustments with IPTW. **p*<0.05 using Pearson’s χ² test.


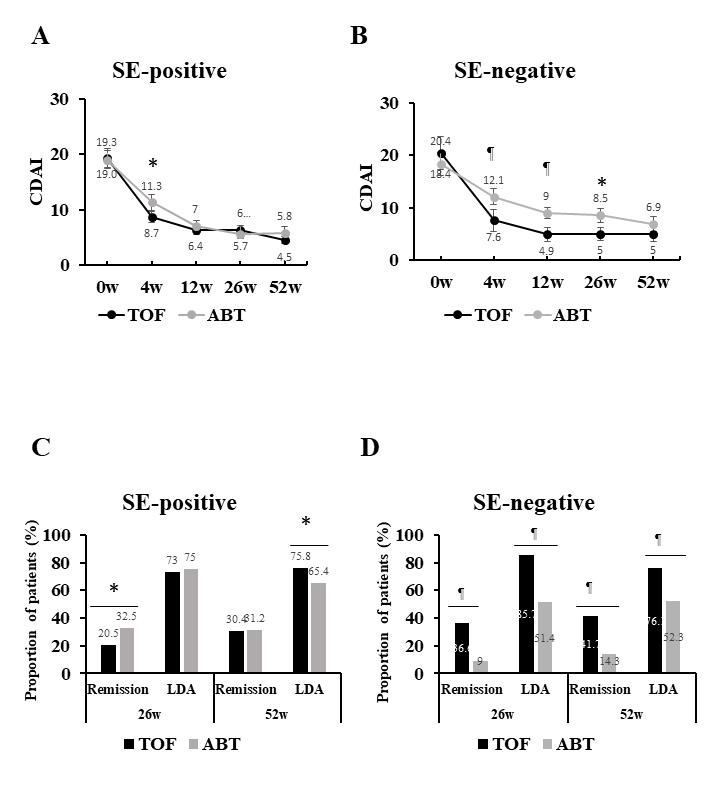


**Supplementary Figure S5. Comparison of tofacitinib (TOF) and abatacept (ABT) groups within each SE category after adjustments with IPTW.** CDAI scores for SE-positive (A) and SE-negative patients (B) are shown over 52 weeks after the start of treatment with TOF or ABT. CDAI scores between the two treatment groups were compared at each time point. The proportion of patients achieving remission and low disease activity (LDA) in SE-positive (C) and SE-negative (D) patients were compared between the two treatment groups by Pearson’s χ² test. Error bars indicate 95% confidence intervals. **p*<0.05, ¶ *p*<0.01 by the Student’s *t*-test. SE, shared epitope.

**Supplementary Table S1. Data collection schedule in this study**

| Variables | baseline | week 4 | week 12 | week 26 | week 52 |
| --- | --- | --- | --- | --- | --- |
| Age | X |  |  |  |  |
| Sex | X |  |  |  |  |
| BMI | X |  |  |  |  |
| Smoking status | X |  |  |  |  |
| Comorbidities | X |  |  |  |  |
| Disease duration | X |  |  |  |  |
| Steinbrocker stage & class | X |  |  |  |  |
| Shared epitope | X |  |  |  |  |
| Rheumatoid factor | X |  |  |  |  |
| Anti-CCP antibody | X |  |  |  |  |
| Therapy for RA at enrollment | X |  |  |  |  |
| TJC | X | X | X | X | X |
| SIC | X | X | X | X | X |
| ESR | X | X | X | X | X |
| CRP | X | X | X | X | X |
| MMP-3 | X | X | X | X | X |
| GH, VAS | X | X | X | X | X |
| EGA, VAS | X | X | X | X | X |
| HAQ-DI | X | X | X | X | X |
| Number of neutrophils | X | X | X | X | X |
| Number of lymphocytes | X | X | X | X | X |
| Hemoglobin | X | X | X | X | X |
| Albumin | X | X | X | X | X |
| AST | X | X | X | X | X |
| ALT | X | X | X | X | X |
| LDL cholesterol | X | X | X | X | X |
| HDL cholesterol | X | X | X | X | X |
| Creatinine | X | X | X | X | X |
| Creatinine phosphokinase | X | X | X | X | X |
| Adverse events | X | X | X | X | X |

An X indicates an outpatient visit for data acquisition. AST, aspartate aminotransferase; ALT, alanine aminotransferase; BMI, body mass index; CCP, cyclic citrullinated peptide; CRP, C-reactive protein; EGA, evaluator’s global assessment of disease activity; ESR, erythrocyte sedimentation rate; GH patient’s global assessment of general health; HAQ-DI, Health Assessment Questionnaire Disability Index; HDL, high density lipoprotein; LDL, low density lipoprotein; MMP-3, matrix metalloproteinase 3; RA, rheumatoid arthritis; SJC, swollen joint count; TJC, tender joint count.

**Supplementary Table S2. Logistic regression model for the estimation of propensity scores for over all patients.**

| Variables | OR and 95%CI | | | |
| --- | --- | --- | --- | --- |
|  | OR | Lower | Higher | *p* value |
| Intercept | 20.791 | 0.032 | 13625.992 | 0.359 |
| Age | 1.022 | 0.997 | 1.049 | 0.0897 |
| Sex, male | 0.938 | 0.391 | 2.248 | 0.886 |
| Duration | 1.020 | 0.996 | 1.044 | 0.106 |
| Height | 0.992 | 0.960 | 1.026 | 0.651 |
| Weight | 1.002 | 0.972 | 1.032 | 0.903 |
| Shared epitope | 0.755 | 0.420 | 1.359 | 0.349 |
| Rheumatoid factor | 1.000 | 1.000 | 1.001 | 0.219 |
| C-reactive protein | 1.005 | 0.852 | 1.186 | 0.950 |
| ESR | 0.990 | 0.975 | 1.004 | 0.170 |
| Number of neutrophils | 1.000 | 1.000 | 1.000 | 0.667 |
| Number of lymphocytes | 1.000 | 0.999 | 1.000 | 0.106 |
| Hemoglobin | 0.892 | 0.702 | 1.132 | 0.374 |
| Albumin | 0.518 | 0.241 | 1.110 | 0.0906 |
| SJC | 1.042 | 0.951 | 1.140 | 0.378 |
| EGA VAS | 0.974 | 0.958 | 0.991 | 2.49x10^-3^ |
| DAS28-ESR | 1.088 | 0.765 | 1.548 | 0.607 |
| HAQ-DI | 1.425 | 0.963 | 2.107 | 0.0764 |
| bDMARD-naïve, yes | 9.509 | 5.362 | 16.864 | 1.30x10^-14^ |
| Anti-CCP antibody positive | 1.359 | 0.603 | 3.060 | 0.459 |

Anti-CCP antibody, anti-citrullinated antibody; bDMARD, biologic disease modifying antirheumatic drug; CI, confidence interval; DAS28-ESR, Disease Activity Score in 28 joints using erythrocyte sedimentation rate; EGA VAS, visual analog scale by evaluator global assessment; HAQ-DI, Health Assessment Questionnaire Disability Index; OR, odds ratio; SJC, swollen joint count.

**Supplementary Table S3. Baseline characteristics of SE-positive patients in TOF and ABT groups before and after IPTW**

|  | Before IPTW | | |  | After IPTW | | |
| --- | --- | --- | --- | --- | --- | --- | --- |
| Variables | TOF (n=135) | ABT (n=122) | *P* value |  | TOF (n=133) | ABT (n=120) | *P* value |
| Age, years | 67.2±11.4 | 70.3±11.1 | 0.022 |  | 69.3±10.7 | 66.9±15.2 | 0.15 |
| Female, n, (%) | 115 (85.2) | 99 (81.1) | 0.48 |  | 105 (78.9) | 100 (83.3) | 0.19 |
| Disease duration, years | 15.2±11.2 | 14.3±12.9 | 0.20 |  | 16.0±12.5 | 14.5±11.9 | 0.33 |
| BMI, kg/m² | 21.8±3.9 | 21.6±3.0 | 0.92 |  | 21.5±3.7 | 21.3±3.2 | 0.68 |
| MTX dose, mg/week | 8.7±2.4 | 8.2±2.6 | 0.34 |  | 8.6±2.2 | 8.7±2.9 | 0.81 |
| Oral corticosteroid dose, mg/day* | 4.2±2.4 | 4.6±3.2 | 0.61 |  | 4.8±2.6 | 4.3±3.1 | 0.42 |
| bDMARD naïve, n (%) | 29 (21.5) | 78 (63.9) | 1.3x10^-11^ |  | 62 (46.6) | 53 (44.2) | 0.58 |
| Number of biologics previously used, n, 1/2/≥3 | 29/41/27/38 | 78/24/8/12 | 1.4x10^-11^ |  | 62/29/17/25 | 53/25/19/23 | 0.60 |
| SJC, 0−28 | 3.7±3.9 | 4.3±4.0 | 0.10 |  | 4.0±3.7 | 3.7±3.7 | 0.63 |
| TJC, 0−28 | 5.3±4.6 | 5.1±4.8 | 0.63 |  | 5.4±4.4 | 4.7±4.7 | 023 |
| GH, VAS, 0−100 mm | 55.6±26.6 | 54.5±24.9 | 0.63 |  | 52.4±26.7 | 53.5±25.5 | 0.74 |
| EGA, VAS, 0−100 mm | 50.2±20.6 | 43.7±19.1 | 0.015 |  | 46.9±18.9 | 44.4±20.0 | 0.31 |
| DAS28-ESR | 4.7±1.5 | 4.8±1.3 | 0.46 |  | 4.7±1.4 | 4.6±1.3 | 0.55 |
| SDAI | 21.6±11.8 | 21.2±11.7 | 0.69 |  | 21.3±11.5 | 20.1±11.0 | 0.39 |
| CDAI | 19.7±10.8 | 19.2±9.9 | 0.77 |  | 19.3±10.5 | 18.3±9.5 | 0.39 |
| ESR, mm/h | 39.5±30.9 | 43.8±31.1 | 0.22 |  | 39.6±30.0 | 40.2±30.6 | 0.88 |
| CRP, mg/dL | 1.9±2.4 | 2.0±2.6 | 0.25 |  | 1.9±2.1 | 1.8±2.4 | 0.70 |
| MMP-3, ng/mL | 216.0±223.8 | 216.6±250.3 | 0.60 |  | 237.1±243.2 | 205.0±231.6 | 0.30 |
| Rheumatoid factor positive, n (%) | 112 (83.0) | 105 (86.1) | 0.61 |  | 106 (80.0) | 103 (85.8) | 0.11 |
| Anti-CCP antibody positive, n (%) | 125 (92.6) | 112 (91.8) | 0.99 |  | 126 (94.7) | 113 (94.1) | 0.76 |
| HAQ-DI | 1.0±0.8 | 1.2±0.8 | 0.17 |  | 1.2±0.9 | 1.2±0.8 | 0.75 |

*Prednisolone equivalent.

Results are expressed as means±SD unless otherwise stated. Comparisons of matched groups were performed using the Student’s *t*-test for continuous variables and Pearson’s χ² test for categorized variables. ABT, abatacept; CCP, cyclic citrullinated peptide; CRP, C-reactive protein; CDAI, Clinical Disease Activity Index; DAS28-ESR, Disease Activity Score in 28 joints using erythrocyte sedimentation rate; GH, patient’s global assessment of general health; HAQ-DI, Health Assessment Questionnaire Disability Index; IPTW, inverse probability of treatment weighting; MTX, methotrexate; RA, rheumatoid arthritis; SE, shared epitope; SDAI, Simplified Disease Activity Index; SJC, swollen joint count; TJC, tender joint count; TOF, tofacitinib.

**Supplementary Table S4. Baseline characteristics of SE-negative patients in TOF and ABT groups before and after IPTW**

|  | Before IPTW | | |  | After IPTW | | |
| --- | --- | --- | --- | --- | --- | --- | --- |
| Variables | TOF (n=52) | ABT (n=61) | *P* value |  | TOF (n=51) | ABT (n=58) | *P* value |
| Age, years | 63.9±11.4 | 71.6±11.2 | 3.2x10^-4^ |  | 66.7±10.6 | 68.05±11.2 | 0.54 |
| Female, n, (%) | 43 (82.7) | 55 (90.1) | 0.37 |  | 45 (88.2) | 54 (93.1) | 0.20 |
| Disease duration, years | 11.5±11.9 | 14.4±13.2 | 0.30 |  | 10.0±12.3 | 13.3±11.2 | 0.14 |
| BMI, kg/m² | 22.7±3.6 | 22.5±4.4 | 0.55 |  | 22.5±3.9 | 23.2±4.2 | 0.36 |
| MTX dose, mg/week | 8.5±2.6 | 8.1±1.8 | 0.37 |  | 8.6±2.6 | 8.3±1.8 | 0.66 |
| Oral corticosteroid dose, mg/day* | 4.8±2.8 | 4.7±2.0 | 0.58 |  | 5.8±3.6 | 4.6±2.1 | 0.19 |
| bDMARD naïve, n (%) | 11 (21.2) | 44 (72.1) | 1.8x10^-7^ |  | 23 (45.1) | 28 (48.3) | 0.68 |
| Number of biologics previously used, n, 1/2/≥3 | 11/19/12/10 | 44/7/4/5 | 1.0x10^-6^ |  | 23/14/8/6 | 28/11/14/5 | 0.22 |
| SJC | 3.9±4.0 | 4.5±3.1 | 0.90 |  | 4.3±4.7 | 3.7±3.3 | 0.36 |
| TJC | 5.8±5.4 | 6.0±5.3 | 0.62 |  | 6.3±5.9 | 4.8±5.1 | 0.14 |
| GH, 0−100 mm | 53.5±29.5 | 51.3±24.5 | 0.66 |  | 50.2±30.3 | 52.8±23.1 | 0.61 |
| EGA, 0−100 mm | 46.4±21.1 | 43.6±15.7 | 0.48 |  | 46.7±21.4 | 45.0±14.5 | 0.63 |
| DAS28-ESR | 4.4±1.5 | 4.9±1.2 | 0.040 |  | 4.6±1.7 | 4.4±1.5 | 0.43 |
| SDAI | 20.9±11.9 | 21.6±9.5 | 0.43 |  | 21.8±13.3 | 19.8.5±9.4 | 0.34 |
| CDAI | 19.7±11.1 | 19.9±8.7 | 0.60 |  | 20.4±12.3 | 18.2±8.8 | 0.29 |
| ESR, mm/h | 29.9±27.3 | 41.5±25.9 | 5.6x10^-3^ |  | 35.7±28.3 | 35.1±29.3 | 0.92 |
| CRP, mg/dL | 1.2±1.5 | 1.7±2.0 | 0.012 |  | 1.4±1.5 | 1.5±2.0 | 0.71 |
| MMP-3, ng/mL | 198.3±209.6 | 216.0±237.7 | 0.48 |  | 245±259.6 | 165.8±205.0 | 0.085 |
| Rheumatoid factor positive, n (%) | 37 (71.2) | 47 (77.0) | 0.62 |  | 33 (64.7) | 34 (58.6) | 0.33 |
| Anti-CCP antibody positive, n (%) | 38 (73.1) | 46 (75.4) | 0.95 |  | 31 (60.8) | 33 (56.9) | 0.67 |
| HAQ-DI | 0.7±0.6 | 1.1±0.8 | 0.031 |  | 0.7±0.7 | 0.9±0.7 | 0.25 |

*Prednisolone equivalent.

Results are expressed as means±SD unless otherwise stated. Comparisons of matched groups were performed using the Student’s *t*-test for continuous variables and Pearson’s χ² test for categorized variables. ABT, abatacept; CCP, cyclic citrullinated peptide; CRP, C-reactive protein; CDAI, Clinical Disease Activity Index; DAS28-ESR, Disease Activity Score in 28 joints using the erythrocyte sedimentation rate; GH, patient’s global assessment of general health; HAQ-DI, Health Assessment Questionnaire Disability Index; IPTW, inverse probability of treatment weighting; MTX, methotrexate; RA, rheumatoid arthritis; SE, shared epitope; SDAI, Simplified Disease Activity Index; SJC, swollen joint count; TJC, tender joint count; TOF, tofacitinib.
